# Case series of rash associated with influenza B in school children

**DOI:** 10.1111/irv.12296

**Published:** 2014-11-10

**Authors:** Danuta M Skowronski, Catharine Chambers, William Osei, Jill Walker, Martin Petric, Monika Naus, Yan Li, Mel Krajden

**Affiliations:** a British Columbia Centre for Disease ControlVancouver, BC, Canada; b University of British ColumbiaVancouver, BC, Canada; c Northern Health AuthorityPrince George, BC, Canada; d National Microbiology LaboratoryWinnipeg, MB, Canada; e University of ManitobaWinnipeg, MB, Canada

**Keywords:** Exanthem, inactivated, influenza, influenza vaccine, influenza-like illness, morbilliform, rash, vaccine

## Abstract

This case series describes morbilliform and other rash presentations among schoolchildren during a March 2014 outbreak of influenza-like illness (ILI) in British Columbia, Canada. Multiplex nucleic acid testing of nasopharyngeal specimens and paired serologic investigations identified that influenza B, characterized as B/Massachusetts/02/2012-like (Yamagata-lineage), was the only viral aetiology and most likely cause of ILI and rash. An association between influenza B and rash has been described infrequently elsewhere, and not previously in North America. Influenza B should be considered in the differential diagnosis of febrile exanthem. Evaluation of the nature, incidence and contributing agent–host–environment interactions, and immunologic mechanisms to possibly explain influenza-associated rash is warranted.

## Introduction

Late-season influenza B activity occurred in Canada during the 2013–2014 season, with circulating viruses predominantly belonging to the B/Yamagata-lineage included in the 2013–2014 trivalent influenza vaccine (TIV). In March 2014, an outbreak of influenza-like illness (ILI) involving the elementary and high school (∼200 students combined) of a rural community (population < 1500) of British Columbia (BC), Canada, was reported, with 15% and 8% of the student populations affected, respectively. Rash associated with ILI was noted in four students, including generalized maculopapular rash in an elementary-school child. Interest in fever associated with rash illness was heightened because of a large measles outbreak occurring simultaneously elsewhere in the province. This case series describes an outbreak of ILI and rash associated with laboratory-confirmed influenza B in schoolchildren.

## Methods

Outbreak investigation was conducted under the authority of the Medical Health Officer, and research ethics board approval was not required. Laboratory testing was conducted according to standard protocols at the BC Public Health Microbiology and Reference Laboratory^1^ that encourage submission of specimens from up to six patients to arrive at ILI outbreak diagnosis. Further specimens were collected to ensure that ILI cases with rash known to the local health unit were included in diagnostic testing. Nasopharyngeal swabs were tested for influenza by reverse-transcription polymerase chain reaction (RT-PCR) and for respiratory viruses by the Respiratory Virus Panel Luminex® assay, which includes targets for influenza A/H3, A/H1 and B; RSV; coronaviruses 229E, OC43, NL63, and HKU1; parainfluenza 1–4; human metapneumovirus A/B; entero/rhinovirus; adenovirus; and bocavirus. Further nucleic acid testing for measles, enterovirus and mumps was conducted. Influenza-positive specimens were sequenced to determine lineage and where possible, virus was isolated in cell culture to determine strain by haemagglutination inhibition (HI) assay. Paired sera were collected, and antibody titres were assessed by HI using live and ether-extracted B/Massachusetts/02/2012-like (Yamagata-lineage) and B/Brisbane/60/2008-like (Victoria-lineage) viruses. Sera were also tested for IgM/IgG to measles, human parvovirus-B19 and rubella. Clinical and epidemiological information was obtained by local public health staff using a standard questionnaire.

## Case series

Six tested students (**C1–C6**) had laboratory-confirmed influenza B infection, including three (**C4–C6**) with localized rash (Tables 1 and 2). One additional student (**E1**) developed generalized rash and was epidemiologically linked through shared classroom exposure to **C1** and **C5** but was RT-PCR negative for influenza. Illness onset dates ranged March 5–12, ages ranged 6–14 years, and 4/7 were female. ILI symptoms did not substantially differ across cases.

**Table 1:** Clinical and epidemiologic features of cases in series

	C1	C2	C3	C4	C5	C6	E1
ILI							
Symptoms	Fever chills Cough Coryza headache Sore throat myalgia Prostration	Fever Cough Coryza Headache Prostration	Fever chills cough Headache Sore throat Myalgia arthralgia Prostration	Fever Cough myalgia Arthalgia Prostration	Fever Cough Coryza Headache Sore throat Myalgia Prostration	Cough Sore throat fatigue	Fever Cough Headache Sore throat Prostration
Other symptoms	Sneeze ↓ appetite Red cheeks Diarrhoea	Sneeze ↓ appetite Conjunctivitis photophobia Tearing	Sneeze ↓ appetite Chest pain Diarrhoea Nausea Vomiting Dizziness	↓ appetite Chest pain Dyspnoea Abdominal pain	Sneeze ↓ appetite Conjunctivitis Photophobia Abdominal pain	None specified	↓ appetite Photophobia Tearing Nausea Vomiting Abdominal pain
Duration of ILI symptoms	10 days	9 days	9 days	8 days	11 days	3 days	11 days
Epidemiological links – shared settings among cases in series							
School	Elementary	Elementary	Elementary	High School	Elementary	High School	Elementary
Grade	—	—	—	C6	—	C4	—
Classroom	C5, E1	—	—	—	C1, E1	—	C1, C5
Household	C2	C1	—	—	—	—	—
Rash							
Affected body part	None	None	None	Localized: Back of hands	Localized: Cheeks nose peri-orbital	Localized: Back of hands	Generalized: sparing palms and soles
Type	NA	NA	NA	Macular non-itchy	Macular itchy	Papular non-itchy	Maculopapular itchy
Features	NA	NA	NA	Followed hot shower	Facial numbness	None specified	Worse with cold air/water
Interval from ILI symptom onset to rash onset	NA	NA	NA	2 days	4 days	0 days	2 days
Duration of rash illness	NA	NA	NA	1 days	4 days	3 days	9 days

ILI, influenza-like illness; NA, not applicable.

**Table 2:** Laboratory findings among cases in series

	C1	C2	C3	C4	C5	C6	E1
Nasopharyngeal specimen – RT-PCR results							
Interval from ILI symptom onset to specimen collection							
	6 days	0 days	7 days	4 days	5 days	2 days	6 days
Influenza	B/Yamagata	B/Yamagata	B/Yamagata	B/Yamagata	B/Yamagata	B/Yamagata	Negative
Enterovirus	Negative	Negative	Negative	Negative	Negative	Negative	Negative
Measles	Negative	Negative	Negative	Negative	Negative	Negative	Negative
Mumps	TND	Negative	TND	Negative	Negative	Negative	Negative
Other RV*	Negative	Negative	Negative	Negative	Negative	Negative	Negative
Characterization of influenza virus isolates – HI assay results							
Strain	TND**	B/Mass**	B/Mass	B/Mass	TND	B/Mass	NA
Paired sera							
Interval from ILI onset to serum collection							
First	14 days	8 days	15 days	12 days	14 days	14 days	14 days
Second	47 days	41 days	40 days	33 days	35 days	35 days	39 days
Influenza serology – inverse HI titre based on ether-extracted virus (geometric mean of duplicate titres)***							
B/Massachusetts/02/2012 (Yamagata-lineage)†							
First	1810	3620	226	320	57	160	1810
Second	905	905	160	226	57	160	1280
B/Brisbane/60/2008 (Victoria-lineage)††							
First	14	7	57	320	320	5	5
Second	5	5	40	160	226	5	7
Influenza serology – inverse HI titre based on live virus (geometric mean of duplicate titres)***							
B/Massachusetts/02/2012 (Yamagata-lineage)†							
First	113	320	10	20	5	20	160
Second	80	80	5	20	5	10	80
B/Brisbane/60/2008 (Victoria-lineage)††							
First	5	5	10	113	160	5	5
Second	5	5	5	40	113	5	5
Other serology							
Measles IgM/IgG							
First	NR/R	NR/R	NR/NR	NR/Inconcl	NR/R	NR/R	NR/R
Second	NR/R	NR/R	NR/Inconcl	NR/NR	NR/R	NR/R	NR/R
Parvovirus B19 IgM/IgG							
First	NR/NR	NR/NR	NR/NR	NR/NR	NR/NR	NR/R	NR/NR
Second	NR/NR	NR/NR	NR/NR	NR/NR	NR/NR	NR/R	NR/NR
Rubella IgG							
First	R	R	R	R	R	R	R
Second	R	R	R	R	R	R	R

RT-PCR, reverse-transcription polymerase chain reaction; TND, test not done; RV, respiratory virus; ILI, influenza-like illness; HI, haemagglutination inhibition; B/Mass, B/Massachusetts/02/2012-like strain; NA, not applicable; NR, non-reactive; R, reactive; Inconcl, inconclusive.

* See text for targets of multiplex respiratory virus panel.

** Sequencing of the haemagglutinin gene from original specimens identified no unusual features and phylogenetic analysis confirmed closest alignment with B/Massachusetts/02/2012-like (clade 2) virus (Genbank numbers: KP083464 and KP083465).

*** Titres < 10 assigned a value of 5.

† B/Massachusetts/02/2012-like (Yamagata-lineage) virus is the recommended reference strain for the 2013–2014 TIV.

†† B/Brisbane/60/2008-like (Victoria-lineage) virus was the recommended reference strain for the 2009–2010 to 2011–2012 TIV and remains the recommended Victoria-lineage reference virus for 2014–2015 quadrivalent influenza vaccine formulations.

**Figure 1:**
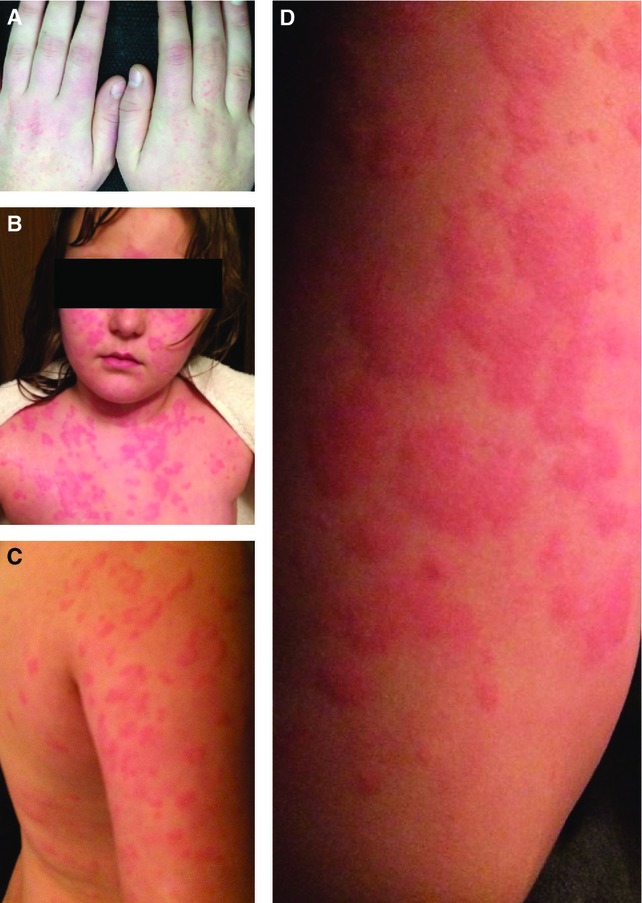
Photographs of rash in association with confirmed influenza B illness. (A) Localized papular non-pruritic rash involving both hands (case C6). (B–D) Generalized maculopapular pruritic rash (case **E1**) including face and chest (B), arms and back (C) and legs (D).

Among the three students with localized rash, two were high-school students in the same grade with erythematous, non-pruritic rash of the back of the hands, sparing the palms, one macular (**C4**) and one papular (**C6**; Figure 1A). The third student with localized rash (**C5**) attended the elementary school and reported facial rash that was erythematous, pruritic, macular and continuous over the cheeks, nose and around the eyes, with conjunctivitis and photophobia.

**E1's** rash was erythematous, pruritic and maculopapular, beginning on the arms and face 2 days after ILI onset with spread to the rest of the body, sparing the palms and soles (Figure 1B–D). There was no oral enanthem. Rash persisted 9 days, worsening with exposure to cold air/water. Tearing and photophobia, without conjunctivitis, and nausea, vomiting, abdominal pain and loss of appetite were accompanying symptoms.

None of the students with rash reported change in diet, detergent, or other products in contact with skin, had an allergic history or comorbidity, or took medications in the week before/after symptom onset to explain rash illness. The one exception may be **C5** who receives daily aspirin prophylaxis for an unrelated medical condition and completed a 5-day course of amoxicillin for the current ILI episode beginning 2 days prior to rash onset; however, rash did not recur with subsequent use of amoxicillin for another indication. All sought medical care; none were hospitalized. All but one received two measles/mumps/rubella (MMR) vaccine doses, with one single-dose recipient. Only **E1** had received the 2013–2014 TIV (inactivated) administered as a single dose 11·5 weeks prior to ILI onset. **E1** had also previously received TIV in 2006, 2007 and 2008.

All six influenza-positive specimens were characterized by RT-PCR as B/Yamagata-lineage; of the four viruses that could be isolated in cell culture, all were characterized as B/Massachusetts/02/2012-like (Table 2). Although a nasopharyngeal specimen collected from **E1** at 6 days post-ILI onset was influenza negative by RT-PCR, HI antibody titres to B/Massachusetts/02/2012-like virus in sera collected from **E1** were comparable to or higher than titres in **C1–C6**. In all cases, titres were lower in paired sera collected 21–33 days after the first serum. This likely reflects the 8- to 14-day delay from ILI onset to first serum collection, at which point infection-induced titres may have already been peaking and fourfold rise (i.e. sero-conversion) could not subsequently be shown. On balance, serologic findings in **E1** are more consistent with recent influenza B infection than prior immunization. In all children, HI titres were higher when using ether-extracted versus live influenza B virus, as expected, but trends were similar. In two children with localized rash (**C4, C5**), titres were similar or higher to the alternate B/Victoria-lineage compared to the B/Yamagata-lineage strain confirmed by PCR and/or culture to be the cause of their current ILI.

No other viral aetiology was identified based on multiplex nucleic acid testing or serology (Table 2).

## Discussion

Here, we describe rash associated with influenza B in schoolchildren during a late-season ILI outbreak. In addition to typical ILI symptoms, three students developed localized rash and one developed generalized morbilliform rash. Six of seven students investigated (including three with localized rash) had laboratory-confirmed influenza B, while the seventh with generalized rash had serologic evidence of infection and was confirmed through epidemiological links to two laboratory-confirmed cases of influenza B in the same classroom (one also with rash). Although amoxicillin may have been a contributing factor in the laboratory-confirmed classmate with localized rash, lack of recurrence with subsequent re-exposure to the same antibiotic argues against allergic aetiology. No co-infection or other viral aetiology was identified in any of the seven ILI cases investigated.

Rash is an uncommon manifestation of influenza. Two prior published reports describe rash with laboratory-confirmed influenza B, both noting morbilliform features: a case report from India in an 11-year-old^2^ and a case series including six children in Germany aged 4–13 years old with ILI and generalized exanthem and enanthem.^3^ Hope–Simpson reported ∼2% and 8%, respectively, of medically attended influenza A and B infections in a British community between 1962 and 1966 had rash, but rash features were not described.^4^ Among 151 patients hospitalized with influenza in Australia in 1982, 4 of 56 (7%) <15 years old (and none ≥15 years old) presented with rash; 3/4 were initially diagnosed as measles, and of these three, two were influenza B.^5^ Among adult Singaporean military recruits, rash (undescribed) was more often associated with influenza A/H3N2 than A(H1N1) pdm09 or influenza B.^6^ Rash associated with influenza A has been variously characterized as petechial,^7^,^8^ macular,^7^ papular,^9^ maculopapular,^10^ reticular^9^ or purpuric^11^,^12^ and has been localized,^9^ multifocal^7^,^8^,^12^ or generalized,^10^ pruritic^9^ and non-pruritic.^7^ Generalized maculopapular rash associated with influenza A(H1N1) pdm09 spared the palms and soles, as was also noted here for influenza B.^10^

While no conclusions can be drawn from a single case, it is interesting that the child with generalized rash was the only one in the current series to have received influenza vaccine. Immunization would not have been a direct cause of rash 3 months later but prior sensitization may nevertheless be relevant. Pre-existing vaccine- or infection-induced antibody might be hypothesized to play a role in rash pathogenesis through the rapid formation of antigen–antibody complexes upon re-exposure. In that regard, it is also interesting that 2 of 3 children with localized rash (and no history of prior influenza immunization) raised substantial antibody titres to previously circulating B/Victoria-lineage virus, suggesting prior infection-induced priming to epitopes shared with the currently infecting B/Yamagata-lineage strain. Antigen and antibody levels at a precise balance may be required for complex formation and in explaining the unusual occurrence and varying nature of influenza-associated rash, particularly late in the season. Ultimately, proposed mechanisms for influenza-associated rash remain speculative. However, prior receipt of the current season's TIV is nevertheless important because it may have attenuated the amount and duration of virus shedding, relevant given the negative RT-PCR result in the child with generalized rash and 6-day delay to nasopharyngeal specimen collection. High titres to B/Massachusetts/02/2012-like virus in that child's sera reinforce the influenza B diagnosis otherwise confirmed through epidemiologic links. Ultimately, however, we cannot rule out other unrecognized aetiologies or contributing factors. Multiplex testing did not identify another viral infection, but the association between influenza and rash does not prove causality; it remains possible that patients in this series were simultaneously infected with an unidentified pathogen or that some other environmental factor contributed.

In conclusion, this is the first report from North America of rash associated with influenza B. Influenza B should be included in the differential diagnosis of febrile exanthem recognizing that, as for influenza A, rash may include varied clinical presentations. Further evaluation of the nature, incidence and contributing agent–host–environment interactions, and immunologic mechanisms to possibly explain influenza-associated rash is warranted.
